# P-Glycoprotein Mediated Efflux Limits the Transport of the Novel Anti-Parkinson's Disease Candidate Drug FLZ across the Physiological and PD Pathological *In Vitro* BBB Models

**DOI:** 10.1371/journal.pone.0102442

**Published:** 2014-07-18

**Authors:** Qian Liu, Jinfeng Hou, Xiaoguang Chen, Gengtao Liu, Dan Zhang, Hua Sun, Jinlan Zhang

**Affiliations:** 1 State Key Laboratory of Bioactive Substance and Function of Natural Medicines, Institute of Materia Medica, Chinese Academy of Medical Sciences & Peking Union Medical College, Beijing, China; 2 Beijing Neurosurgical Institute, Capital Medical University, Beijing, China; Indian Institute of Integrative Medicine, India

## Abstract

FLZ, a novel anti-Parkinson's disease (PD) candidate drug, has shown poor blood-brain barrier (BBB) penetration based on the pharmacokinetic study using rat brain. P-glycoprotein (P-gp) and breast cancer resistance protein (BCRP) are two important transporters obstructing substrates entry into the CNS as well as in relation to PD neuropathology. However, it is unclear whether P-gp and BCRP are involved in low BBB permeability of FLZ and what the differences of FLZ brain penetration are between normal and Parkinson's conditions. For this purpose, *in vitro* BBB models mimicking physiological and PD pathological-related BBB properties were constructed by C6 astroglial cells co-cultured with primary normal or PD rat cerebral microvessel endothelial cells (rCMECs) and *in vitro* permeability experiments of FLZ were carried out. High transepithelial electrical resistance (TEER) and low permeability for sodium fluorescein (NaF) confirmed the BBB functionality of the two models. Significantly greater expressions of P-gp and BCRP were detected in PD rCMECs associated with the lower *in vitro* BBB permeability of FLZ in pathological BBB model compared with physiological model. In transport studies only P-gp blocker effectively inhibited the efflux of FLZ, which was consistent with the *in vivo* permeability data. This result was also confirmed by ATPase assays, suggesting FLZ is a substrate for P-gp but not BCRP. The present study first established *in vitro* BBB models reproducing PD-related changes of BBB functions *in vivo* and demonstrated that poor brain penetration of FLZ and low BBB permeability were due to the P-gp transport.

## Introduction

As the main functional interface between the circulatory system and brain, the blood-brain barrier (BBB) is a major challenge for effective delivery of therapeutics to the brain [1], [2]. Approximately 98% of small molecule drugs and all large molecule neurotherapeutics are barely able to cross BBB [3], unless they are actively taken up into the brain. For this reason, most drugs presently in clinical use for CNS therapy are lipophilic compounds with molecular weight less than 500 Da. However, a variety of small lipophilic therapeutics which were predicted to permeate the brain were also obstructed by the BBB due to the presence of drug efflux transporters localized on surface of the cerebral microvessel endothelial cells [4]. P-glycoprotein (P-gp) and breast cancer resistance protein (BCRP), both widely expressed in murine and human brain [5], are two important drug pumps limiting substrates across BBB [6] as well as involved in the neuropathology of Parkinson's disease (PD) [7].

**Figure 1:**
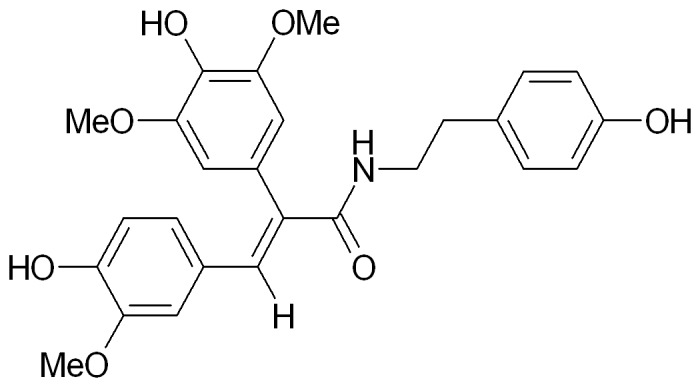
The chemical structure of FLZ.

A novel anti-PD candidate drug, FLZ, formulated as N-2-(4-hydroxy-phenyl)-ethyl]-2-(2,5-dimethoxy-phenyl)-3-(3-methoxy-4-hydroxy-phenyl)-acrylamide (Figure1), showed strong neuroprotective effects in experimental PD models *in vitro* [8], [9] and *in vivo* [10], [11]. Pharmacokinetic study demonstrated FLZ could enter the brain, therapeutic targets for PD, rapidly with no lag time [6]. However, as a lipophilic small molecule compound (molecular weight 458.2 Da), the penetration ratio of FLZ to brain was only 0.073 [6], indicating that FLZ alone had limited access to the brain. Therefore, we hypothesize drug pumps such as P-gp and BCRP may be involved in the transport of FLZ across the BBB and result in the low BBB permeability. To test this hypothesis, the interactions of FLZ with both P-gp and BCRP were examined through *in vitro* BBB models.

Previous studies have strongly indicated the different level of BBB efflux transporters during the progression of neurodegenerative disorders [12]. However, it is not known whether pathology-related changes in the BBB functions *in vivo* are reproducible in the primary culture of rat cerebral microvessel endothelial cells (rCMECs) isolated from animals under Parkinson's conditions. In this study, physiological and pathological BBB models were constructed from primary normal and PD rCMECs under contact co-culture with C6 astroglial cells on the Transwell supports. The differences in BBB properties and permeability of FLZ in the two models were tested. The contributions of P-gp and BCRP to FLZ transport were evaluated by examining the influence of zosuquidar and Fumitremorgin C (FTC), the P-gp and BCRP specific inhibitors, on the BBB permeability of FLZ in the physiological and PD pathological *in vitro* BBB models. Moreover, to further confirm the results, drug efflux transporter membrane ATPase assays had been done.

## Materials and Methods

### Reagents and antibodies

FLZ, a white powder with 99% purity (by HPLC), was synthesized by the Department of Medicinal Chemistry, the Institute of Materia Medica. All reagents used in the study were purchased from Sigma (St. Louis, MO, USA), unless otherwise indicated. Collagenase/dispase and basic fibroblast growth factor (bFGF) were obtained from Roche Molecular Biochemicals (Indianapolis, IN, USA). Transwell-Clear (polyester) permeable supports, 0.4 µm pore size, were acquired from Corning (Acton, MA, USA). Percoll was obtained from Pharmacia (Uppsala, Sweden). Human P-gp and BCRP membrane and the ATPase assay kit were purchased from BD Gentest Discovery Labware Inc. (BD Biosciences, Woburn, MA, USA). Endothelial Cell Medium (ECM) was obtained from Sciencell (San Diego, CA, USA). Fetal bovine serum (FBS), Horse serum (HS), Ham's F-10 nutrient mixture and Hanks Balanced Salt Solutions (HBSS) were obtained from Gibco BRL (Grand Island, NY, USA). Rabbit anti von Willebrand factor (vWF), Mouse anti glial fibrillary acidic protein (GFAP), Mouse anti GAPDH, Mouse anti BCRP and Rabbit anti P-gp were obtained from Santa Cruz (Delaware Avenue, CA, USA). Texas Red goat anti-rabbit IgG antibody, Texas Red goat anti-mouse IgG antibody, Alexa Fluor goat anti-rabbit IgG antibody, Alexa Fluor goat anti-mouse IgG antibody and Texas Red-X phalloidin and 4′,6-diamidino-2-phenylindole (DAPI) dihydrochloride nuclear stain were purchased from Invitrogen (Carlsbad, CA).

### Animal and treatment

Wistar rats were obtained from Vital River Laboratories (Beijing, China). All experiments were performed in accordance with the guidelines established by the National Institutes of Health for the care and use of laboratory animals and were approved by the Animal Care Committee of the Peking Union Medical College and Chinese Academy of Medical Sciences. All surgery was performed under sodium pentobarbital anesthesia, and all efforts were made to minimize suffering.

### Experimental design

**Figure 2:**
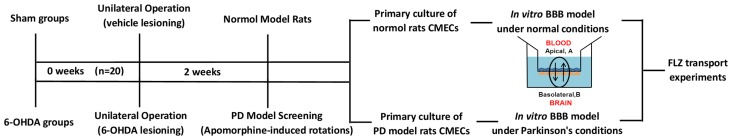
Experimental design. Rats were randomly divided into sham and 6-hydroxydopamine (6-OHDA) groups (n = 20 per group) which were injected with vehicle or 6-OHDA (10 µg) unilaterally into the SN, respectively. The successfully PD model rats were screened by apomorphine (0.5 mg/kg) two weeks later after unilateral operation. Then primary normal and PD rCMECs were isolated from brain microvessel fragments of sham and PD model rats and contact co-culture with C6 astroglial cells to construct physiological and PD pathological *in vitro* BBB models, respectively. The transport of FLZ was measured on the physiological and pathological *in vitro* BBB models in the absence or presence of transporters specific inhibitors.

In our study (Figure 2), rats were randomly divided into sham and 6-hydroxydopamine (6-OHDA) groups (n = 20 per group) which were injected with vehicle or 6-OHDA (10 µg) unilaterally into substantia nigra (SN), respectively. The successful PD model rats were screened by apomorphine (0.5 mg/kg) two weeks later after unilateral operation. Then, primary normal and PD rCMECs were isolated from brain microvessel fragments of sham and PD model rats respectively and used to construct *in vitro* BBB models under normal and Parkinson's conditions. Physiological and pathological BBB models were established in transwell supports constructed from cultured C6 astroglial cells contacting with normal or PD rCMECs, and concentration-dependent transepithelial transport of FLZ was measured. To better study the contribution of P-gp and BCRP to FLZ permeability *in vitro*, we measured the translocation of FLZ in the absence or presence of zosuquidar and FTC, the transporters specific inhibitors, in the physiological and pathological BBB models.

### Unilateral 6-OHDA lesioning procedure

Adult male Wistar rats weighing 250–290 g were randomly divided into sham and 6-hydroxydopamine (6-OHDA) groups (n = 20 per group). Rats were injected under pentobarbital anesthesia (35 mg/kg) with 6-OHDA unilaterally into the SN. 6-OHDA was freshly dissolved with 0.9% saline containing 0.02% ascorbic acid just before use and protected from light to minimize oxidation. 10 µg 6-OHDA at a dose of 2 µg/ml was injected into in each location at a flow rate of 0.5 µl/min. (coordinates: bregma −5.2, lateral 2.2 and ventral 7.8 mm). After the injection, the needle was left in place for an additional 10 min before being slowly withdrawn from the brain. The sham-operated rats were injected with the vehicle alone into the same locations as their respective experimental groups.

### Behavioral tests (Apomorphine-induced rotations)

The drug-stimulated rotational response was measured two weeks after the 6-OHDA SN lesioning. Following peritoneal injection of R-apomorphine hydrochloride solution (0.5 mg/kg), on every SN lesion model rat the rotation test was performed and recorded for 45 min. Model animals that rotated significantly (more than 280 times contralateral turns/45 min) in the two weeks after the lesion were taken as the successful PD model rats according to former reports [13].

### Isolation of normal and PD model rat cerebral microvessel endothelial cells (rCMECs)

Isolation of rCMECs was based on a modified protocol as described [14]. Brains were removed from sham and PD model rats and stored in Dulbecco's modified Eagle's medium (DMEM) on ice. Meninges were carefully removed from forebrains and gray matter was mashed with forceps and minced into small pieces of approximately 1 mm^3^, then digested with 1 mg/ml type II collagenase and 50 U/ml Dnase I in DMEM for 1.5 h in 37°C shaker (200 rpm). Cold DMEM was added to the homogenate and centrifuged at 1000 g, 4°C for 8 min. The pellet was resuspended in 20% bovine serum albumin (BSA) and centrifuged at 1000 g, 4°C for 20 min. The microvessels obtained in the pellet were further digested with 1 mg/ml collagenase/dispase and 25 U/ml DNase I in DMEM for 1 h in 37°C shaker (200 rpm). The microvessels were separated on a 33% continuous Percoll gradient, collected and washed twice in DMEM before plating on 35 mm collagen IV/fibronectin-coated Petridish (both 0.1 mg/ml). rCMECs cultures were maintained in the Endothelial Cell Medium (ECM) supplemented with 100 µg/ml heparin and 4 µg/ml puromycin [15] and maintained in humidified air containing 5% (v/v) CO_2_ at 37°C in incubator. After 3 days, the culture medium was replaced and puromycin was removed from ECM. When the cultures reached 80% confluency, the purified normal and PD model rCMECs were passaged by a brief treatment with trypsin (0.05%, w/v) –EDTA (0.02%, w/v) solution, and used to construct physiological and pathological *in vitro* BBB models, respectively.

### Rat C6 astroglial cells cultures

Rat C6 astroglial cells were obtained from the American Type Culture Collection (ATCC) and maintained in Ham's F-10 media supplemented with 15% HS, 2.5% FBS and antibiotics (penicillin 100 U/ml, streptomycin 100 µg/ml). Rat C6 astroglial cells were grown on 75 cm^2^ culture flasks and passaged using 0.1% trypsin–EDTA solution in a humidified 37°C incubator with 5% CO_2_.

### Immunostaining

Morphological observations were taken under the Phase-Contrast microscopy (Olympus IX70, Tokyo, Japan). To confirm the purity of normal and PD rCMECs, the expressions of a specific endothelial cell marker (vWF), transport proteins P-gp and BCRP were identified. Normal and PD rCMECs monolayers cultured on fibronectin-coated 35 mm cell culture dishes were fixed at room temperature (RT) in PBS containing 4% paraformaldehyde (20 min) and then permeabilized for 10 min with 0.1% TritonX-100, respectively. Cells were blocked with 3% BSA and incubated with anti-vWF antibody (1∶100 dilution) overnight. The secondary antibody (FITC-conjugated goat anti-rabbit IgG) was applied for 1 h, after washing three times with PBS. To counterstain cell, DAPI was used according to the manufacturer's instructions. Images were examined by PerkinElmer UltraVIEW VoX system (PerkinElmer Life Sciences Inc., MA, USA). As with rat C6 astroglial cells in the polyester membrane of the Transwell inserts, the expression of a relatively specific astrocyte cell marker (GFAP) was determined according to the same protocol.

### Western blot

Cells were harvested and lysed in nondenaturing lysis buffer (Applygen Technologies, Beijing, China). Equal quantities (40 µg of protein) of cell extract were resolved by 10% SDS–PAGE, and the resolved proteins were electrophoretically transferred to PVDF membrane and blocked with 5% fat-free dry milk in TBST for 2 h at room temperature. Then respective primary antibodies were added in 5% milk TBST and incubated overnight at 4°C and incubated with horseradish peroxidase-conjugated secondary antibodies for 2 h at room temperature and washed three times before detection. The blot was developed with LAS4000 enhanced chemiluminescence system (GE Healthcare, Buckinghamshire, UK) and the densities of the bands were determined using Gel-Pro Analyzer 4.0 software.

### Construction of *in vitro* BBB models under normal and Parkinson's conditions

To construct physiological and pathological *in vitro* BBB models, rat C6 astroglial cells (2.5×10^5^ cells/cm^2^) were seeded on the bottom side of the collagen-coated polyester membrane of Transwell-Clear permeable supports (polyester membranes, 0.4 µm pore size, 6.5 mm diameter) for overnight, allowing the cells to adhere to the membrane firmly. Then normal or PD model rCMECs (5×10^5^ cells/cm^2^) were seeded on the upper side of the inserts placed in the well of the 24-well culture plates. The BBB models were maintained in ECM with media replacement occurring every other day until the monolayers reached confluency.

### Electron microscopy

Processing was conducted as previously described [14]. Briefly, Cells grown on the membrane were fixed with 3% paraformaldehyde in cacodylate buffer (pH 7.5) for 30 min at 4°C. After washing with 0.1 M PBS, the membranes of the Transwell inserts with the cells on the two sides were removed from their support and postfixed in 1% osmium tetraoxide (OsO_4_) for 30 min. After several subsequent washes with PBS, the membranes were dehydrated with gradient alcohol and embedded in Epon resin. Randomly selected ultrathin sections were stained with uranyl acetate and lead citrate and examined using a transmission electron microscope (H-7650, HITACHI, Tokyo, Japan).

### Transendothelial electrical resistance (TEER) level measurements

The TEER values were measured using a Millicell-ERS voltohmmeter (Millipore, Inc. USA) every two days. Final resistances (Ω/cm^2^) were calculated after subtracting the resistance of collagen-coated control membranes.

### Measurement of fluorescein permeability

The flux of sodium fluorescein (NaF) across the BBB models was determined as previously described [16]. Prior to transport studies, culture media from the top and bottom compartments were replaced with HBSS. 100 µg/ml Na-F (MW: 376 Da) was added to the upper inserts of the Transwell system. At 30, 60, 90 and 120 min, 50 µl samples were taken from the acceptor compartment and immediately replaced with fresh buffer. The concentrations of the fluorescein in samples from the upper to lower compartments were determined by fluorescence multiwell plate reader (excitation wavelength: 485 nm; emission wavelength: 535 nm). Based on the rate of influx of sodium fluorescein into the bottom compartment, permeability coefficients were calculated.

### *In vitro* drug transport experiments

FLZ transport experiments were carried out in 24-well Transwells in the BBB models. The permeability of FLZ was measured in two directions: apical-to-basolateral (A–B) and basolateral-to-apical (B–A). Before the transport experiments, TEER value across each insert was measured and after washing with prewarmed (37°C) Hank's balanced salt solution (HBSS), the cultures were preincubated with fresh HBSS either alone or containing the P-gp inhibitor zosuquidar (5 µM) or BCRP inhibitor FTC (10 µM) at the indicated concentrations for 30 min at 37°C. The experiment was initiated by replacing the medium in the donor compartment (either apical or basolateral) with fresh HBSS containing FLZ (1, 5, 10 µM) or the mixture of 10 µM FLZ and P-gp or BCRP inhibitors. The plates were kept under culture conditions throughout the experiment. Samples (50 µl) were taken from the acceptor compartment at predetermined times (30, 60, 90, 120 and 150 min) after addition of FLZ to the donor side. The volume sampled (50 µl) was immediately replaced with fresh HBSS. Samples from the permeability studies were analyzed on Agilent Technologies 6410 Triple Quad LC/MS system (Agilent Corporation, MA, USA). The apparent permeability (Papp) coefficients in cm/s were calculated according to the following formula equation as previously described [17].
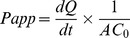



Where dQ/dt is the permeability rate, which is the slope of a plot of the cumulative receiver concentration by time, A is the membrane surface area (0.33 cm^2^), and C_0_ is the initial donor concentration. 
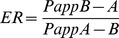



The efflux ratio (ER), defined as the ratio of Papp in the B–A direction to the Papp in the A–B direction, was used to estimate the magnitude of P-gp or BCRP-mediated efflux.

### ATPase assay

The P-gp or BCRP-associated ATPase activity was measured by BD Gentest ATPase kit. The assay was carried out in white opaque 96-well multiplates in triplicate. Recombinant human P-gp or BCRP membrane (5 mg/ml) was quickly thawed and diluted to 1 mg/ml with assay buffer. Sodium orthovanadate (Na_3_VO_4_) was used as a ATPase inhibitor. Various concentrations of FLZ or inhibitors(5 µM zosuquidar, 10 µM FTC or 100 µM Verapamil)diluted with assay buffer were incubated in 20 µg (20 µl) diluted recombinant human Pgp or BCRP membrane at 37°C for 5 minutes. Initiate reactions by adding 20 µl of 15 mM Mg^2+^ATP to all wells. At this point, each Pgp reaction contains 5 mM ATP. Mix briefly on a plate shaker or by gently tapping the plate. Incubate for 40 minutes at 37°C. Luminescence initiated by ATP detection buffer. After incubated at 37°C for 20 min to allow luminescent signal to develop, the untreated white opaque 96-well multiplates was read on luminometer (SpectraMax M5, molecular devices, USA). The changes of relative light units (ΔRLU) were determined by comparing Na_3_VO_4_-treated samples with FLZ and inhibitors combination-treated groups.

### Statistical analysis

Data were expressed as means ± SD. Statistical analysis of the data was performed using the one-way ANOVA. *P*<*0.05* was considered to be significant statistically. Statistical tests were carried out using SPSS 13.0 statistical software.

## Results

### Isolation and characterization of normal and PD rCMECs primary culture

**Figure 3:**
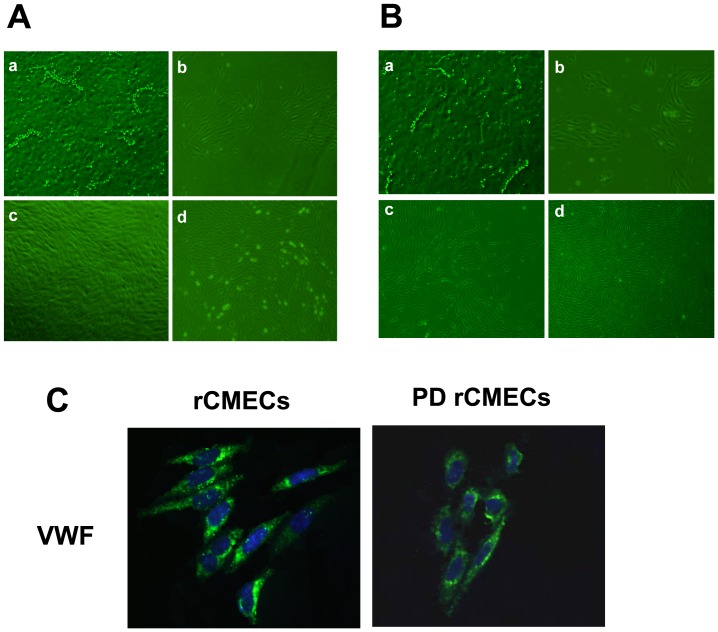
Phase photomicrograph and characterization of rCMECs primary culture by immunofluorescence microscopy. Phase-contrast micrographs of normal (A) and PD (B) rCMECs in primary culture 0 (a), 3 (b), 5 (c) and 7 (d) days after inoculation. In primary culture 3 days the initial outgrowth morphology of normal (Ab) and PD (Bb) rCMECs colonies were similar to an elongated swirling pattern. As the cultures matured and the cells in the colonies multiplied in primary culture 5 days, normal rCMECs (Ac) became elongated and grew in non-overlapping continuous monolayers, but similar states in PD rCMECs (Bc) have not yet been observed. In 7 days after inoculation the normal (Ad) and PD (Bd) rCMECs formed typical “classic cobblestone” morphology and appeared to contact-inhibition property when the monolayers were confluent. The time of PD rCMECs growing into confluent monolayers was longer than rCMECs in spite of cultured at approximate cell density. Panel C are the immunofluorescent images of rCMECs cultures probed for vWF (green) and DAPI nuclear stain (blue). Both normal and PD rCMECs express endothelial cells specific marker. Scale bar  = 100 µm.

Two-step enzyme digestions with one-step gradient centrifugation were used to isolate normal (Figure 3Aa) and PD brain (Figure 3Ba) microvessel fragments. Samples were initially seeded at approximate cell density to both normal and PD rCMECs obtained by the puromycin purification method [18], in primary culture 3 days after treated with 4 µg/ml puromycin, the initial outgrowth morphology of normal (Figure 3Ab) and PD (Figure 3Bb) rCMECs colonies were similar to an elongated swirling pattern described before [19]. As the cultures matured and the cells in the colonies multiplied in primary culture 5 days, normal rCMECs (Figure 3Ac) became elongated and grew in non-overlapping continuous monolayers, but similar states in PD rCMECs (Figure 3Bc) have not yet been observed. In 7 days after inoculation the normal (Figure 3Ad) and PD (Figure 3Bd) rCMECs formed typical “classic cobblestone” morphology and appeared to contact-inhibition property when the monolayers were confluent and positive immunostaining for vWF, a specific marker of endothelial cells (Figure 3C).

### Expression of drug efflux transporters P-gp and BCRP in normal and PD rCMECs

To investigate the effects of FLZ on the growth of normal and PD rCMECs monolayers, a cell proliferation assay was performed. Treatment with FLZ at the concentrations of 1 to 50 µM, P-gp inhibitor 5 µM zosuquidar and BCRP inhibitor 10 µM FTC respectively did not effect upon the viability of either normal or PD rCMECs after the treatment for 24 h, assessed by MTT (Figure S1). Therefore, in all following experiments, we used FLZ (1, 5 and 10 µM), zosuquidar (5 µM) and FTC (10 µM) as standard treatment.

**Figure 4:**
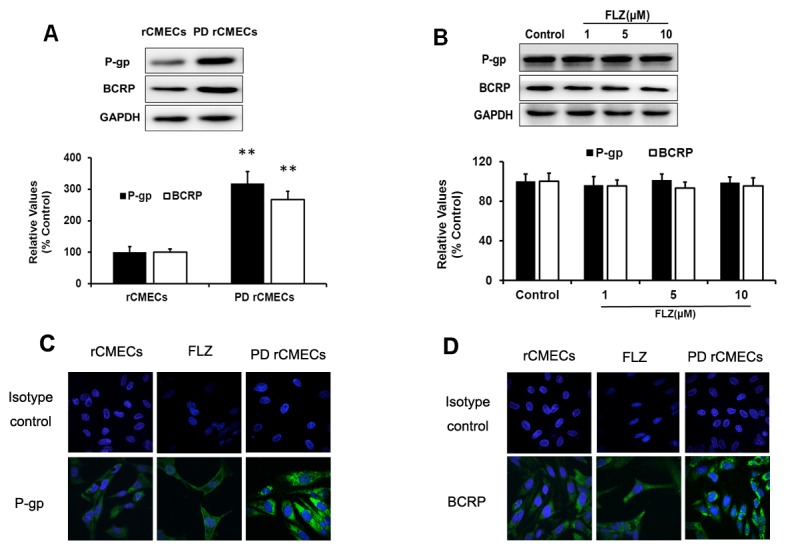
Western blot and confocal microscopy analysis of the expression of P-gp and BCRP in rCMECs. The relative level of the proteins was determined by densitometry. PD rCMECs showed a significant increase of P-gp and BCRP level relative to normal rCMECs detected by Western blot (A). Results are expressed as mean ± SD (n = 3). **P<*0.01*, significantly different from normal rCMECs group. No difference in P-gp and BCRP levels of rCMECs treated with 10 µM FLZ for 24 h (B). A Similar tendency could be observed by immunofluorescence of P-gp (C) and BCRP (D) staining. Bar  = 20 µm.

The levels of drug efflux transporters P-gp and BCRP in normal and PD rCMECs in the absence or presence of FLZ were determined by Western blot (Figure 4A and B) and Immunofluorescent staining (Figure 4C and D). No difference of expression in P-gp and BCRP was observed between the control group and FLZ-treated group (Figure 4B, C and D). However, P-gp and BCRP level was significantly increased in PD rCMECs group compared with normal rCMECs group (Figure 4A, C and D).

### Characterization of the *in vitro* BBB models

**Figure 5:**
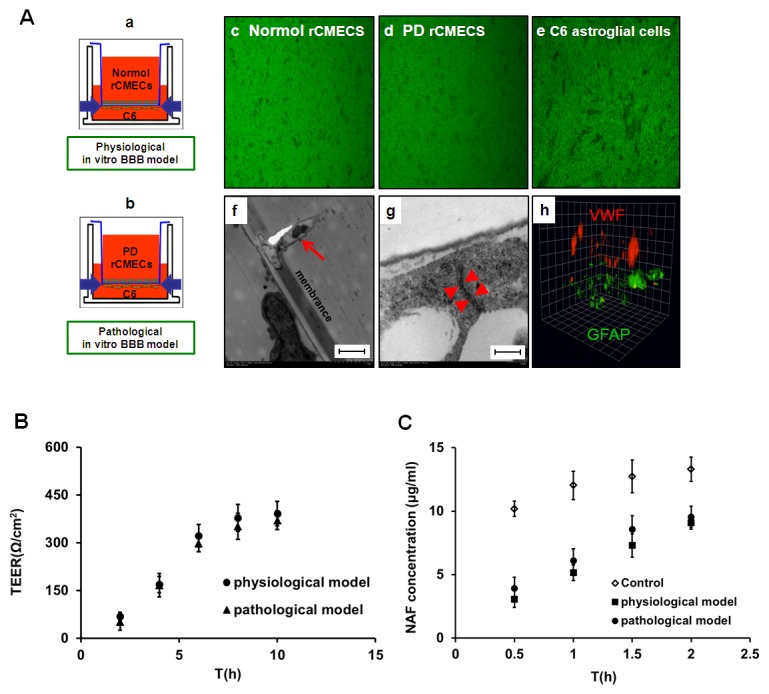
Characterization of the *in vitro* BBB models. Ten days after establishing the physiological (Aa) and PD pathological (Ab) *in vitro* co-culture BBB models, normal rCMECs (Ac), PD rCMECs (Ad) and C6 astroglial cells (Ae) in the polyester membrane of the Transwell inserts grew in non-overlapping continuous monolayers and most endothelial cells became elongated and assumed a swirling pattern. Foot processes of C6 astroglial cells (Af, arrowhead) growing upside down transmigration through collagen-coated inserts with membrane pores 0.4 µm in diameter were observed by transmission electron microscopy. Bar (Af) = 20 µm. The tight junctions (Ag, arrows) bound adjacent endothelial cells together in co-cultures were detected at high magnification (×150000). Bar (g) = 2 µm. The expression and distribution of rCMECs and C6 astroglial cells in the Transwell membrane was visualized by immunofluorescent 3D images probed for vWF (red) and GFAP (green) (Ag, on the apical cell side). 1unit  = 11.82 µm. Panel B: Transendothelial electrical resistance (TEER, expressed as Ω/cm^2^) of the *in vitro* BBB models. Panel C: Endothelial permeability coefficient for sodium fluorescein (NaF Papp, expressed in 10^−6^ cm/s) of the physiological and pathological *in vitro* BBB models. High TEER (B) and low paracellular Papp of NaF (C) confirmed the BBB functionality of the two models for the *in vitro* drug permeability experiment. All data are expressed as the means ± SD (n = 5).

The physiological (Figure 5Aa) and pathological (Figure 5Ab) *in vitro* BBB models were realized in a Transwell system using normal and PD rCMECs on the collagen-coated inserts co-culture with C6 astroglial cells as described in “Materials and Methods” section. Ten days after establishing the co-cultures, normal (Figure 5Ac) or PD rCMECs (Figure 5Ad) and C6 astroglial cells (Figure 5Ae) in the polyester membrane of the Transwell inserts grew in non-overlapping continuous monolayers and most endothelial cells became elongated and assumed a swirling pattern.

The co-culture models were examined by immunostaining probed for vWF (a marker for endothelium) and GFAP (a marker for astroglial cells). The expression and distribution of rCMECs and C6 astroglial cells in the polyester membrane were visualized by cofocol microscope (Figure 5A). As shown in the immunofluorescent 3D images, immunostaining for vWF (red) and GFAP (green) in the membrance of transwell was observed (Figure 5Ah). Correspondingly, the foot processes of C6 astroglial cells growing upside down transmigration through collagen-coated inserts with membrane pores 0.4 µm in diameter were observed at the in contact co-cultures (Figure 5Af. arrowhead).

Detailed ultrastructural examination by electron microscopy could clearly identify the adjacent endothelial cells linked by tight junctions (TJs). Therefore, we observed the TJs at the interface between two neighboring rCMECs grown on the inner chamber membrane of transwell inserts after 10 days in culture by high magnification (×150000). When rCMECs were cultured in contact with C6 astroglial cells, endothelial cells formed a superficial layer around the polyester (apical surface) and the tight junction (Figure 5Ag. arrow) appears in sections as points of cell-cell contact where the exterior membrane leaflets of the neighboring cells appeared to fuse, occluding in consequence the paracellular space.

In addition, the characterization of the physiological and PD pathological *in vitro* BBB models was compared by measurement of TEER and endothelial permeability coefficient for NaF. Although PD rCMECs mono-culture (absence C6) TEER values(268±19 Ω/cm^2^) were significantly lower than TEER values of normal rCMECs mono-culture (312±23 Ω/cm^2^), the resistance of PD co-culture model increased up to 371±29 Ω/cm^2^ at day 10, no significant difference compared to the co-culture models under normal conditions (392±32 Ω/cm^2^) (Figure 5B), suggesting an increase tightness of endothelial monolayers when co-cultured with C6 astroglial cells. The paracellular permeability of rCMECs as measured by the influx rate of water-soluble small marker sodium fluorescein also revealed similar results to TEER. The presence of C6 astroglial cells in either physiological or pathological models dramatically decreased the flux of the tracer (Figure 5C). The low paracellular Papp (expressed as 10^−6^ cm/s) of NaF in the two co-culture models (1.92±0.19 and 2.18±0.26, respectively) verified the establishment of an impermeable barrier under normal and Parkinson's conditions.

### Directional transport of FLZ across the physiological and PD pathological *in vitro* BBB models

**Figure 6:**
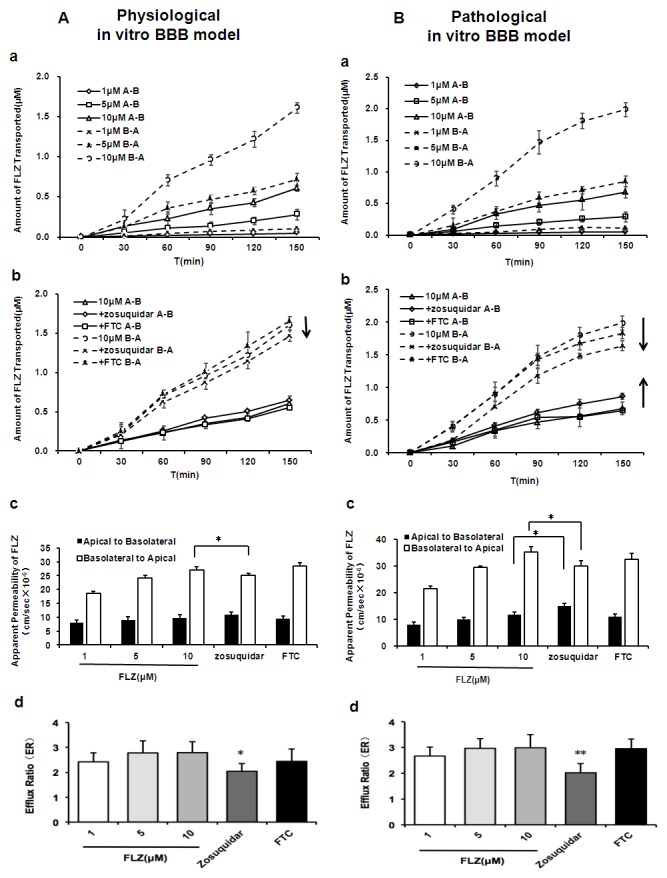
Directional transport of FLZ across physiological (A) and PD pathological (B) BBB models. Transport was measured in the absence or presence of P-gp inhibitor zosuquidar or BCRP inhibitor FTC with time up to 2.5 h. The apical-to-basolateral (A–B) and basolateral-to-apical (B–A) transepithelial flux of various concentrations of FLZ (1, 5, 10 µM) was assessed in physiological (Aa) and pathological (Ba) BBB models, respectively. Papp B–A transport was significantly higher than those for A–B transport at each FLZ concentration (Ac and Bc), indicating the presence of an efflux pump to remove FLZ from within cell membranes. Higher efflux ratio and lower BBB penetration of FLZ were showed under pathology model (Ad and Bd). To examine the contribution of P-gp and BCRP to FLZ transport, permeability of FLZ at initial concentrations of 10 µM in the absence and presence of 5 µM zosuquidar to block P-gp or 10 µM FTC to inhibit BCRP (Ab and Bb) were measured. Only the P-gp inhibitor zosuquidar effectively inhibited efflux of FLZ across the two BBB models, and the black arrows indicate the direction of change in FLZ transport caused by addition of zosuquidar (Ac and Bc). The magnitude of P-gp or BCRP-mediated efflux was estimated by the efflux ratio (ER), defined as the ratio of Papp B–A to the Papp A–B. Upon specific blocking of P-gp using zosuquidar resulted in significantly lower efflux ratio of FLZ in either physiological (Ad) or pathological (Bd) BBB models. However, the efflux ratio between BCRP inhibitor and inhibitor-free group were not significantly different (Ad and Bd). All data are expressed as the means ± SD (n = 3). *P<*0.05*, significantly different from each corresponding control.

The rCMECs expressing efflux pumps is a commonly used tool to study efflux transporter-mediated drug transport. To examine the impact of P-gp or BCRP interactions on FLZ permeability in the *in vitro* BBB model under normal and Parkinson's conditions, bidirectional transport of FLZ across BBB was assessed in the established physiological and pathological BBB models, respectively. The experiments were performed either without inhibitor, in the presence of zosuquidar to block P-gp or FTC to inhibit BCRP. Both the apical-to-basolateral (A–B) and basolateral-to-apical (B–A) fluxes of FLZ across rCMECs cell layers were linear with time up to 2.5 h under all conditions (Figure 6). When no inhibitor was applied, the transport of various concentrations of FLZ (1, 5, 10 µM) through the physiological (Figure 6A) and pathological (Figure 6B) BBB models occurred in both A–B and B–A directions, and Papp B–A transport were significantly higher than those for A–B transport at each FLZ concentration under all conditions (Figure 6Aa, Ac, Ba and Bc), suggesting the presence of efflux pumps to remove FLZ from within cell membranes. In addition, FLZ (1, 5, 10 µM) showed higher efflux ratio in the pathological model compared to physiological BBB model (2.65, 2.97, 2.99 compared to 2.31, 2.70, 2.80, respectivelly) (Figure 6Ad and Bd, Table S1), indicating that the BBB permeability of FLZ may lower under Parkinson's conditions.

To evaluate the contribution of P-gp and BCRP to FLZ transport, bidirectional permeability of FLZ at initial concentration of 10 µM in the absence and presence of zosuquidar or FTC was measured in the two models, respectively (Figure 6Ab and Bb). Only the P-gp inhibitor zosuquidar effectively inhibited P-gp mediated efflux of FLZ were observed across different BBB models, such that Papp A–B permeability (expressed as 10^−6^ cm/s) of FLZ was increased from 11.82±0.92 to 14.92±1.03 in pathological model (Figure 6Bc, Table S1) and the Papp B–A permeability of FLZ was significantly reduced from 27.13±1.01 to 25.02±0.76 in physiological BBB model (Figure 6Ac, Table S1) and 35.38±1.97 to 30.15±1.92 in pathological model (Figure 6Bc, Table S1) in zosuquidar-treated group compared to untreated cells. Moreover, upon specific blocking of P-gp using zosuquidar increased intracellular accumulation of FLZ in rCMECs in a dose-dependent manner (Figure S2) and resulted in significantly lower efflux ratio of FLZ in the BBB models from either normal (Figure 6Ad, Figure S2, Table S1) or Parkinson's disease rats (Figure 6Bd, Table S1). However, BCRP inhibitor, 10 µM FTC, did not affect Papp values for either A–B or B–A transport significantly (Figure 6Ac and Bc, Table S1). Taken together, these data indicate that FLZ is transported by P-gp but not BCRP, and P-gp is likely involved in limiting FLZ penetration that across the BBB under normal and Parkinson's conditions.

### ATPase assays

**Figure 7:**
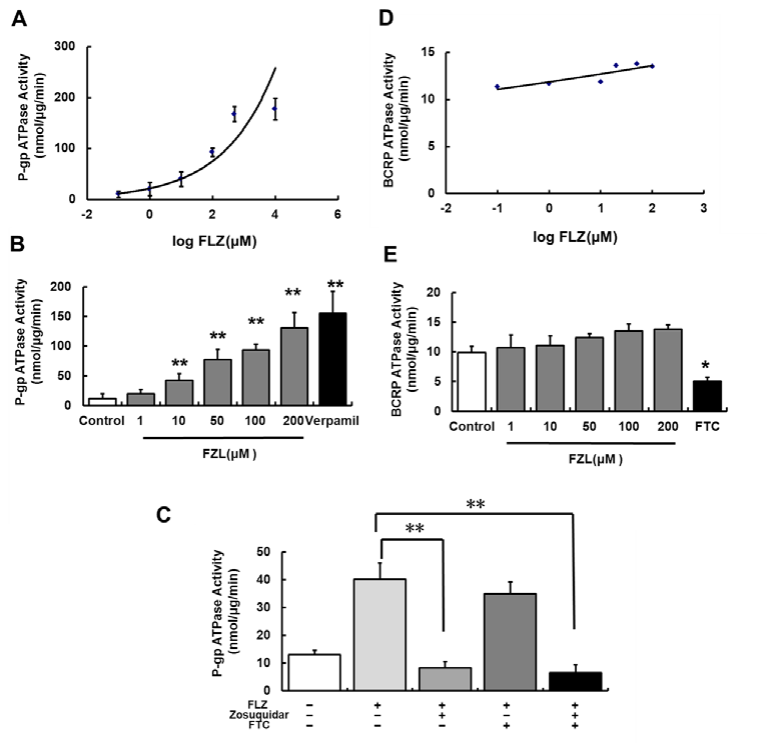
Membrane ATPase assays of P-gp and BCRP activity. Drug efflux transporter ATPase activity was examined in P-gp (A and B) and BCRP (D and E) membrane preparations following various concentrations of FLZ exposure and specific drug efflux transport inhibitors. 200 µM Verapamil was used as a positive control in P-gp ATPase assays. Only the P-gp membranes produced a clear concentration dependent response to FLZ. Results are expressed as mean ± SD (n = 3). Inset in panel C shows the P-gp mediated ATPase activity examined in the presence of 10 µM FLZ and 5 µM zosuquidar (P-gp inhibitor) or/and 10 µM FTC (BCRP inhibitor). All data are expressed as the means ± SD (n = 3). *P<*0.05*, **P<*0.01*, significantly different from FLZ alone.

In order to further prove the substrate specificity of FLZ for the efflux pumps, the interactions between FLZ and P-gp or BCRP were evaluated by using both cell- and membrane-based assays. Cellular accumulation of rhodamine-123 (Rh123, P-gp marker substrates) and Doxorubicin (DOX, a cosubstrate of P-gp and BCRP [20]) was assessed to identify potential interactions of FLZ with P-gp and BCRP in rCMECs (data not shown). A concentration dependent increase in Rh123 and DOX accumulation was observed following FLZ treatment. However, no significant difference was observed in DOX accumulation by FLZ in the presence of zosuquidar blocking P-gp, suggesting that P-gp, but not BCRP, involved in the efflux of FLZ from endothelial cells. Furthermore, the results obtained from the cellular accumulation were confirmed by examining the effect of FLZ on ATPase activity. The efflux function of transporters is coupled to ATP hydrolysis by the ATPase which could be activated in the presence of transporter substrates or modulators. Drug efflux transporter membrane ATPase assays were analyzed following exposure to various concentrations of FLZ or/and specific drug efflux transport inhibitors (Figure 7). Of the two drug efflux transporter membrane preparations examined, only the P-gp membranes produced a clear concentration dependent response to FLZ with an estimated EC_50_ value of 107.14±10.37 µM (Figure 7A) (the positive control verapamil ED_50_ = 12 µM according to the manufacturer's recommendations), whereas FLZ did not stimulate BCRP ATPase activity (Figure 7D and E). To further confirm the specificity of FLZ in the membrane ATPase assay, studies were performed in the presence of zosuquidar or/and FTC, a selective P-gp or/and BCRP inhibitor. In P-gp ATPase studies, 10 µM FLZ produced a significant increase in ATPase activity compared to control group. The increased ATPase activity observed in the FLZ treatment group was completely abolished by addition of 5 µM zosuquidar. In contrast, the addition of BCRP inhibitor, 10 µM FTC, had no effect on FLZ response in P-gp ATPase assay (Figure 7C). Together, these results indicate that FLZ is a substrate for P-gp but not BCRP.

## Discussion

In the present study, we used primary normal and PD rCMECs to reconstitute the *in vitro* BBB models for evaluating the contribution of P-gp and BCRP to FLZ penetration across the physiological and PD pathological BBB. Our study represents the first to compare normal rCMECs with PD rCMECs isolated from Parkinson animals following 6-OHDA SN lesion which has never been formally isolated before. The present work strongly suggested the possibility of reproducing 6-OHDA-related BBB functional differences *in vitro*. It is possible that the increased function of transporters associated with PD model seen in this study might be a consequence of an irritability response to eliminate neurotoxins such as 6-OHDA. Previous *in vivo* study also demonstrated that the apparent increase of P-gp immunoreactivity and dysfunction of the BBB was associated with 6-OHDA-induced DA neuron loss [21]. However, it is still unclear whether PD-related changes in the BBB functions *in vivo* could be reproduced in the primary cultured rCMECs isolated from animals following 6-OHDA lesioning. Our results confirm this correlation based on the slower proliferation and lower TEER values which may be associated with the BBB dysfunction and the overexpression of drug pumps which were observed both in primary PD rCMECs and the animal model (Liu et al., unpublished data). The data also suggest that the different transporter level may contribute to the dissimilarity in BBB permeability for drug pumps substrate under normal and Parkinson's condition.

Since 1978, when the first *in vitro* BBB model was successfully established by monocultures of rCMECs [22], several models constructed from cultured cells have been developed. Now *in vitro* reconstituted BBB models have played an important role in studying various aspects of the BBB [14], including the structural and functional organization of barrier complex [23] and the barrier regulation mechanisms [24], especially extensively used for *in vitro* prediction of the BBB permeability of drugs [1]. Well characterized evidence demonstrates the inductive role of glial cells or astrocyte-conditioned media during the development and maintenance characteristics of BBB *in vitro* [25], [26], [27], [28]. The most common and widely utilized BBB model co-cultures is the brain endothelium on permeable supports together with astrocytes or glial cells [29]. C6 astroglial, which has been widely tested, is a cell line derived from a rat glioma and secretes soluble factors inducing BBB properties in brain microvessels [30], [31] and is effective in tightening the interendothelial junctions when co-cultured with rCMECs [32]. In the present study, to assess FLZ transport through the BBB, we established the syngenic *in vitro* BBB co-culture model using rCMECs and C6 astroglial cells from the same species in close opposition on the underside of Transwell polyester membrane (Figure 5Aa), which was visualized by 3D-immunofluorescence images of co-culture filter (Figure 5Ah) and morphological distribution of the foot processes of C6 through membrane pores (Figure 5Af. arrowhead) determined by electron microscopy. Previous studies have shown abnormalities in BBB and P-gp function following damage to the SN [21]. However, existing *in vitro* BBB models have not been able to directly simulate the BBB under Parkinson's conditions. To obtain the model mimics physiological and pathological BBB properties, PD rat cerebral microvessel endothelial cells were incorporated in co-culture setting (Figure 5Ab). And electron microscopy of the complete TJs complex between adjacent endothelial cells (Figure 5Ag) further revealed the integrity of the co-culture models. The concentration-dependent transepithelial permeability and directional transport of FLZ in the physiological (Figure 6Ac) and pathological (Figure 6Bc) BBB models were shown according to the approximately 3-fold higher Papp B–A transport than A–B transport at each concentration. Moreover, FLZ showed higher efflux ratio and lower BBB permeability (Figure 6Ad and Bd) under pathology model, associated with the overexpression of transporters in PD BBB model (Figure 4).

Current theories propose that the use of specific inhibitors of P-gp and BCRP allows us to study the single contribution of two efflux transporters to the translocation of the tested substrates [33]. Therefore, zosuquidar and FTC, the corresponding well-known specific inhibitors of murine and human P-gp and BCRP, were used in FLZ *in vitro* transport experiment, respectively. By studying P-gp-mediated transport in the presence of zosuquidar, we found a significantly decrease in the efflux ratio for FLZ across the physiological (Figure 6Ad) and pathological (Figure 6Bd) *in vitro* BBB models, respectively. However, blocking BCRP with FTC, no significant reduce effect on efflux ratio of FLZ transport was observed either in physiological (Figure 6Ad) or pathological (Figure 6Bd) BBB model. The results indicated that FLZ is mainly transported by P-gp but not BCRP *in vitro* BBB either under normal or Parkinson's conditions. In addition, evidences supporting FLZ as a substrate for P-gp was observed in cellular accumulation of Rh123 (data not shown) and in the P-gp ATPase assay (Figure 7).

We further compared the *in vitro* transport data of FLZ with previously generated *in vivo* brain penetration results. The brain distribution studies showed that brain-to-plasma ratio of FLZ in the group treated with P-gp inhibitor zosuquidar increased about 20.8-fold at 10 min post-dose, with no significant increase inhibition of BCRP [6]. Results of the *in vivo* microdialysis study also showed that less brain penetration of FLZ was detected in the PD model rats compared to the normal group (Hou et al., unpublished data), which agrees with our observation from the *in vitro* BBB models (Figure 6). The increasing level of P-gp in PD condition may contribute to the obvious dissimilarity.

In conclusion, the results indicate that poor brain penetration of FLZ and low BBB permeability are due to the P-gp transport system in the brain. The data also highlights that 6-OHDA-related barrier properties of rCMECs are retained *in vitro*. From the remarkable agreement between the *in vitro* and *in vivo* data, the BBB models we established from normal and 6-OHDA induced Parkinson's disease rats provide a useful tool for research on physiological/pathological BBB *in vitro*. The present study also generates important information for clinical application of FLZ and further structural transformation for rational drug design to avoid P-gp mediated drug efflux.

## Supporting Information

Figure S1 **The cytotoxicity of FLZ (A) and inhibitors (B) in rat cerebral microvessel endothelial cells.** rCMECs were exposed to the indicated concentration of FLZ (A) and transport inhibitors zosuquidar and FTC for 24 h. Each point represents the mean ± SD of three determinations. Each experiment was performed in three times.
(TIF)

Figure S2 **The dose response of the P-gp inhibitor zosuquidar impact FLZ permeability across the** ***in vitro*** **BBB model.** The apical-to-basolateral (A–B) and basolateral-to-apical (B–A) transepithelial flux of 10 µM FLZ was assessed in the absence or presence of P-gp inhibitor zosuquidar (2.5, 5, 10 µM) with time up to 2.5 h (A). Zosuquidar (2.5, 5, 10 µM) effectively inhibited efflux of FLZ (Papp B–A) across the physiological BBB model in a dose response manner, and the Papp A–B permeability of FLZ was increased only by addition of 10 µM zosuquidar (A and B). The magnitude of P-gp or BCRP-mediated efflux was estimated by the efflux ratio (ER), defined as the ratio of Papp B-A to the Papp A-B. Upon specific blocking of P-gp using zosuquidar resulted in significantly lower efflux ratio of FLZ across BBB models in a dose response manner(c). All data are expressed as the means ± SD (n = 3). *P<*0.05*, ^#^ *P*<*0.05*, **P<*0.01*, significantly different from each corresponding control.
(TIF)

Table S1 **Bidirectional transport of FLZ across physiological and PD pathological BBB models.** The apical-to-basolateral (A–B) and basolateral-to-apical (B–A) transepithelial flux of various concentrations of FLZ (1, 5, 10 µM) was assessed in physiological and pathological BBB models, respectively. To examine the contribution of P-gp and BCRP to FLZ transport, permeability of FLZ at initial concentrations of 10 µM in the absence and presence of 5 µM zosuquidar to block P-gp or 10 µM FTC to inhibit BCRP were measured. Only the P-gp inhibitor zosuquidar effectively inhibited efflux of FLZ across the two BBB models, the Papp A–B permeability (expressed as 10^−6^ cm/s) of FLZ was increased from 11.82±0.92 to 14.92±1.03 in pathological model and the Papp B–A permeability of FLZ was significantly reduced from 27.13±1.01 to 25.02±0.76 in physiological BBB model (p = 0.0459) and 35.38±1.97 to 30.15±1.92 in pathological model (p = 0.0178) in zosuquidar-treated group compared to untreated cells. Upon specific blocking of P-gp using zosuquidar resulted in significantly lower efflux ratio of FLZ in the BBB models from either normal or Parkinson's disease rats. However, the efflux ratio between BCRP inhibitor and inhibitor-free group were not significantly different. All data are expressed as the means ± SD (n = 3). *P<*0.05* significantly different from each corresponding control.
(TIF)
