# Supplementary figures and images for: P-Glycoprotein Mediated Efflux Limits the Transport of the Novel Anti-Parkinson's Disease Candidate Drug FLZ across the Physiological and PD Pathological In Vitro BBB Models
Source: PLoS One. 2014 Jul 18;9(7):e102442. doi: 10.1371/journal.pone.0102442 (PMC4103815; doi:10.1371/journal.pone.0102442)

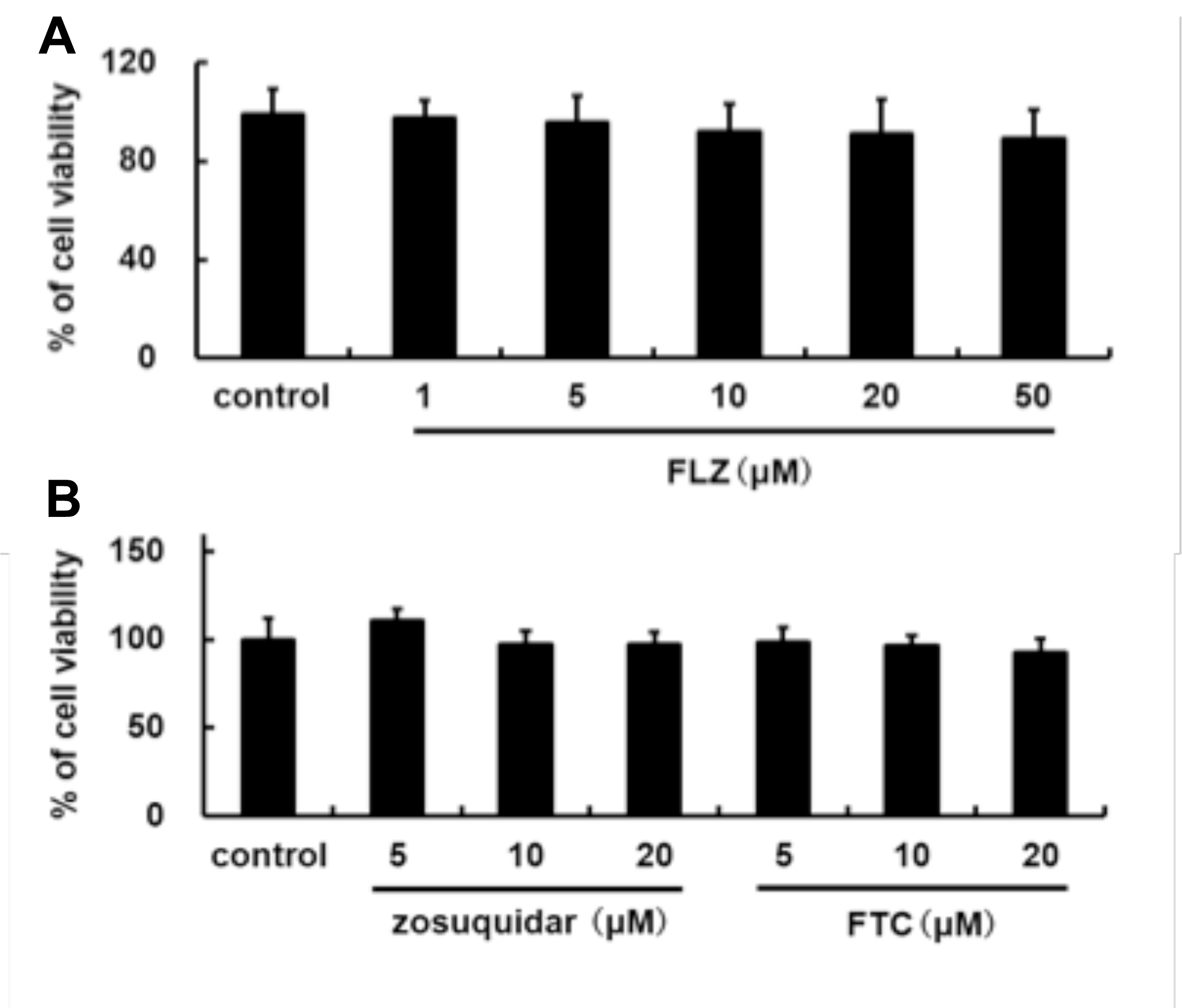

Supplement: Figure S1 — The cytotoxicity of FLZ (A) and inhibitors (B) in rat cerebral microvessel endothelial cells. rCMECs were exposed to the indicated concentration of FLZ (A) and transport inhibitors zosuquidar and FTC for 24 h. Each point represents the mean ± SD of three determinations. Each experiment was performed in three times. (TIF) [file pone.0102442.s001.tif]

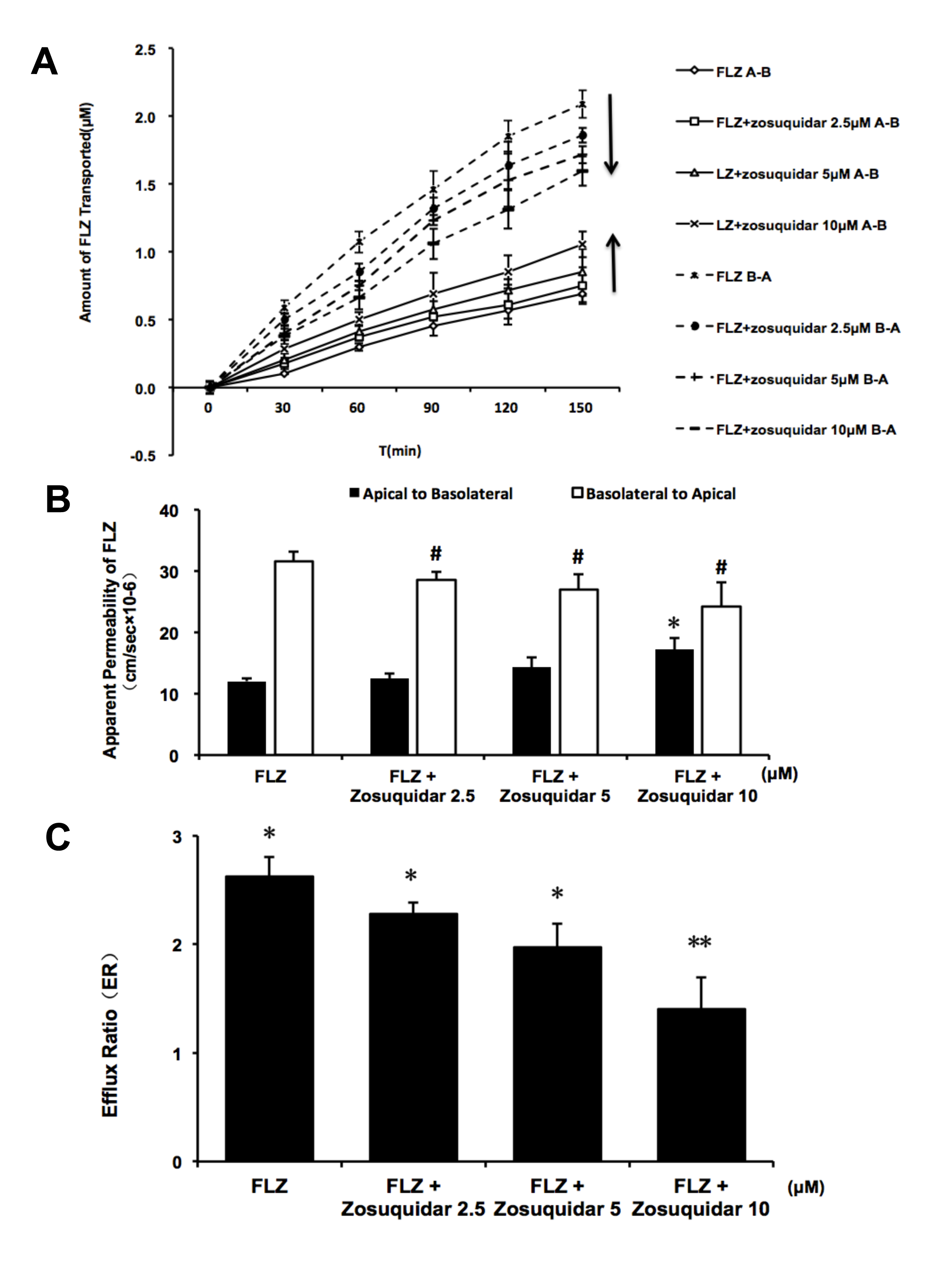

Supplement: Figure S2 — The dose response of the P-gp inhibitor zosuquidar impact FLZ permeability across the in vitro BBB model. The apical-to-basolateral (A–B) and basolateral-to-apical (B–A) transepithelial flux of 10 µM FLZ was assessed in the absence or presence of P-gp inhibitor zosuquidar (2.5, 5, 10 µM) with time up to 2.5 h (A). Zosuquidar (2.5, 5, 10 µM) effectively inhibited efflux of FLZ (Papp B–A) across the physiological BBB model in a dose response manner, and the Papp A–B permeability of FLZ was increased only by addition of 10 µM zosuquidar (A and B). The magnitude of P-gp or BCRP-mediated efflux was estimated by the efflux ratio (ER), defined as the ratio of Papp B-A to the Papp A-B. Upon specific blocking of P-gp using zosuquidar resulted in significantly lower efflux ratio of FLZ across BBB models in a dose response manner(c). All data are expressed as the means ± SD (n = 3). *P<0.05, # P<0.05, **P<0.01, significantly different from each corresponding control. (TIF) [file pone.0102442.s002.tif]

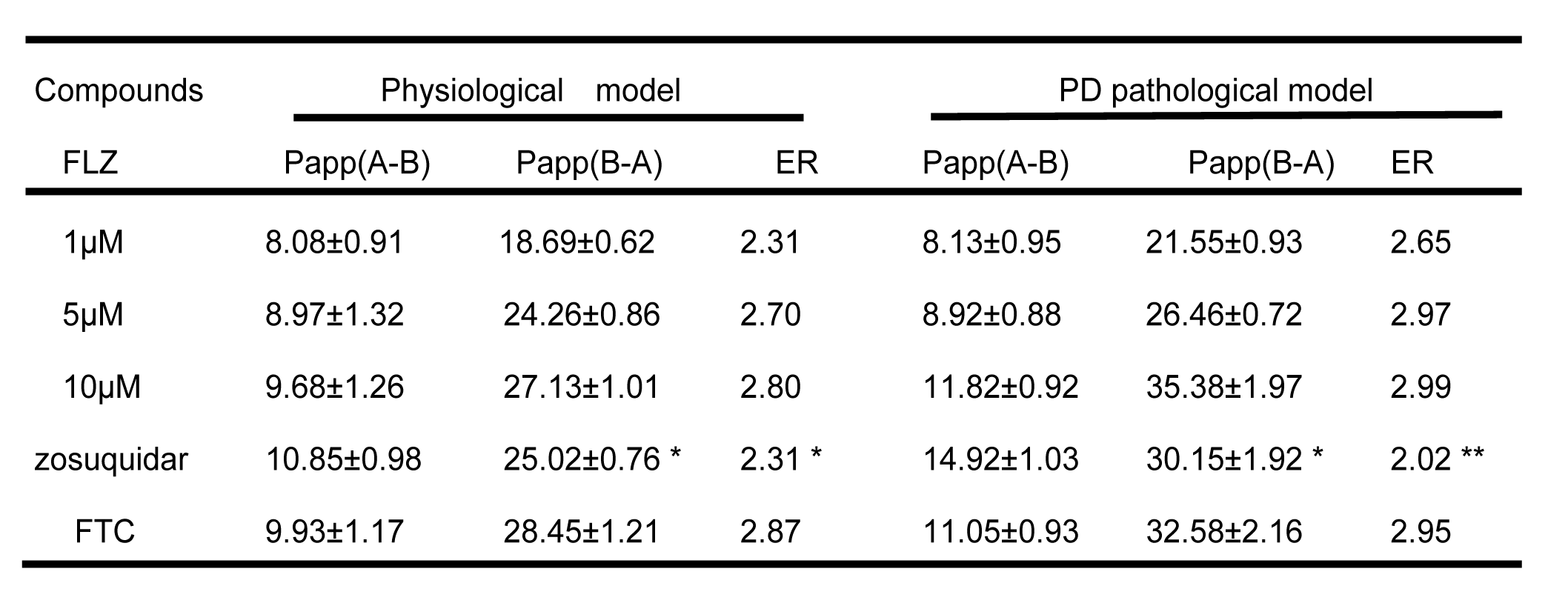

Supplement: Table S1 — Bidirectional transport of FLZ across physiological and PD pathological BBB models. The apical-to-basolateral (A–B) and basolateral-to-apical (B–A) transepithelial flux of various concentrations of FLZ (1, 5, 10 µM) was assessed in physiological and pathological BBB models, respectively. To examine the contribution of P-gp and BCRP to FLZ transport, permeability of FLZ at initial concentrations of 10 µM in the absence and presence of 5 µM zosuquidar to block P-gp or 10 µM FTC to inhibit BCRP were measured. Only the P-gp inhibitor zosuquidar effectively inhibited efflux of FLZ across the two BBB models, the Papp A–B permeability (expressed as 10−6 cm/s) of FLZ was increased from 11.82±0.92 to 14.92±1.03 in pathological model and the Papp B–A permeability of FLZ was significantly reduced from 27.13±1.01 to 25.02±0.76 in physiological BBB model (p = 0.0459) and 35.38±1.97 to 30.15±1.92 in pathological model (p = 0.0178) in zosuquidar-treated group compared to untreated cells. Upon specific blocking of P-gp using zosuquidar resulted in significantly lower efflux ratio of FLZ in the BBB models from either normal or Parkinson's disease rats. However, the efflux ratio between BCRP inhibitor and inhibitor-free group were not significantly different. All data are expressed as the means ± SD (n = 3). *P<0.05 significantly different from each corresponding control. (TIF) [file pone.0102442.s003.tif]
